# Late-life intermittent fasting decreases aging-related frailty and increases renal hydrogen sulfide production in a sexually dimorphic manner

**DOI:** 10.1007/s11357-021-00330-4

**Published:** 2021-03-06

**Authors:** Yoko O. Henderson, Nazmin Bithi, Christopher Link, Jie Yang, Rebecca Schugar, Natalia Llarena, J. Mark Brown, Christopher Hine

**Affiliations:** 1 Department of Cardiovascular and Metabolic Sciences, Cleveland Clinic Lerner Research Institute, 9500 Carnegie Avenue, Cleveland, OH 44195 USA; 2 Reproductive Endocrinology and Infertility, Cleveland Clinic Women’s Health Institute, Cleveland, OH 44195 USA

**Keywords:** Aging, Intermittent fasting, Frailty, Hydrogen sulfide, Cognition, Sexual dimorphism

## Abstract

Global average life expectancy continues to rise. As aging increases the likelihood of frailty, which encompasses metabolic, musculoskeletal, and cognitive deficits, there is a need for effective anti-aging treatments. It is well established in model organisms that dietary restriction (DR), such as caloric restriction or protein restriction, enhances health and lifespan. However, DR is not widely implemented in the clinic due to patient compliance and its lack of mechanistic underpinnings. Thus, the present study tested the effects of a somewhat more clinically applicable and adoptable DR regimen, every-other-day (EOD) intermittent fasting, on frailty in 20-month-old male and female C57BL/6 mice. Frailty was determined by a series of metabolic, musculoskeletal, and cognitive tasks performed prior to and toward the end of the 2.5-month dietary intervention. Late-life EOD fasting attenuated overall energy intake, hypothalamic inflammatory gene expression, and frailty in males. However, it failed to reduce overall caloric intake and had a little positive effect in females. Given that the selected benefits of DR are dependent on augmented production of the gasotransmitter hydrogen sulfide (H_2_S) and that renal H_2_S production declines with age, we tested the effects of EOD fasting on renal H_2_S production capacity and its connection to frailty in males. EOD fasting boosted renal H_2_S production, which positively correlated with improvements in multiple components of frailty tasks. Therefore, late-life initiated EOD fasting is sufficient to reduce aging-related frailty, at least in males, and suggests that renal H_2_S production capacity may modulate the effects of late-life EOD fasting on frailty.

**Supplementary Information:**

The online version contains supplementary material available at 10.1007/s11357-021-00330-4.

## Introduction

The global population over age 65 is expected to nearly double from 8.5 to 16.7% by the year 2050 [1], largely due to advancements in medicine and paradigm shifts in public health policies. While this scenario has obvious personal and societal benefits, it does not come without its disadvantages. Notably, the extension of the median lifespan does not inherently confer the extension of disease-free healthspan [2]. Aging is one of the strongest risk factors for debilitating disorders, such as metabolic syndrome, cardiovascular diseases [3], and neurocognitive deterioration [4]. Thus, preventing aging-related declines in health and limiting the occurrence of these maladies in an ever-expanding aged population is of great importance.

Older adults over age 65 often suffer from not one, but several of these aging-related deficits concurrently, which have negative implications for their quality of life and lifespan. Chronological age (i.e., actual age in years) does not always predict survivorship/mortality; therefore, the concept of frailty was introduced to explain the heterogeneity of health status in the aged (i.e., the biological age; [5, 6]). Frailty is characterized by sex-dependent loss of physiological reserve [7–10], therefore increased susceptibility to various internal and or external stressors leading to poor health outcomes or death. Recently developed frailty indices for mice and rats [11–15] are clinically relevant and predict rodent mortality in a similar manner as clinical frailty indices for humans [11, 14, 16]. However, while often including general behavior tasks, they usually lack hippocampal-dependent memory testing as one of the frailty criteria [11, 17, 18]. For instance, the study that extensively investigated the effects of life-long every-other-day (EOD) intermittent fasting on over 200 different aging-related phenotypes in mice did not include any hippocampal-dependent measurements [19]. Inclusion of the cognitive tests in assessing rodent frailty is critical, given that the aging brain often develops cognitive degeneration which is typically manifested as Alzheimer’s [4], Parkinson’s [20], and Huntington’s diseases [21], as well as general non-specific memory decline. Therefore, the present study utilized a variety of behavioral assessments that are commonly included in the previously developed rodent frailty indices, as well as utilizing the cognitive tests, notably hippocampal-dependent memory tests.

One possible intervention to conquer brain aging and frailty in humans is the adaptation of dietary restriction (DR). DR, such as caloric restriction (CR) and/or sulfur amino acid restriction (e.g., methionine restriction; MetR) without malnutrition is one of the well-established anti-aging interventions that extends health and lifespan in various model organisms, such as yeast, worms, fruit flies, fish, and rodents [22–33]. Importantly, recent studies showed that DR is beneficial for lifespan extension and/or averting frailty in non-human primates [34–42]. Studies in humans also showed that long-term CR is beneficial for a multitude of frailty components. For instance, long-term CR in humans decreased body mass index [43, 44], memory impairments [45], oxidative stress [46], and triiodothyronine [43, 47] and enhanced cardiometabolic systems [44, 47].

However, studies examining the effects of long-term CR on human lifespan, particularly in older adults, have been difficult to execute due to patient adherence to chronic CR [48, 49] and the need for constant medical and scientific oversight to ensure the daily caloric intake and guidelines are safely followed and adhered to [43, 50–53]. In addition, safely implementing CR in older adults may be difficult, as they tend to exhibit anorexia and/or malnutrition [54–56]; therefore, long-term CR may not be a desirable anti-aging intervention in older adults.

Given these limitations, the relatively implementable yet effective DR regimen, intermittent fasting (IF), has gained substantial attention as an alternative method to continuous, chronic CR [57, 58]. Some find IF, or also referred to as time-restricted feeding [59], easier to follow, particularly in older adults. Specifically, participants (≥ 67 years of age) in the study by Conley et al. (2018) [60] were able to successfully implement this dietary regimen for six months [60]. There are several types of IF regimens, including the 5:2 diet [61], every-other-day (EOD) fasting [62, 63], and variations in timed feeding throughout a 24-hour (hr) period [64]. In EOD fasting, animals and/or participants are subjected to no food intake for a day (i.e., 24 hr) followed by food intake ad libitum (AL) for the next 24 hr, which was reported to be safe, tolerable, and produce comparable metabolic outcomes as a continuous CR protocol in humans [65]. Thus, the major difference between IF and continuous CR is that IF does not require daily caloric restriction to drive DR effects.

The beneficial effects of IF on metabolism and diseases and its cellular and molecular responses have been reviewed extensively elsewhere [66]. Notably, EOD fasting in rodents augments (1) lifespan [19, 67–70], (2) neuronal protection against chemical insults [71], (3) cardiovascular responses [19, 72], (4) hippocampal morphology, (5) proteome profile, and function [73], (6) metabolic flexibility [19], and (7) motor coordination [19, 74]. Likewise, EOD fasting decreases the number and/or incidence of tumors [19] and the hippocampal oxidative damage [73] in adult and aging rodents. Also, studies that compared the effects of EOD fasting and continuous CR in adult mice showed that both dietary regimens are comparably effective to enhance glucose metabolism/insulin sensitivity [75] and to protect against aging-related neurobehavioral deficits (i.e., decreased locomotion and memory impairments; [76]). Therefore, these findings suggest EOD fasting initiated in late-life may enhance health and lifespan, at least in rodents.

**Fig. 1:**
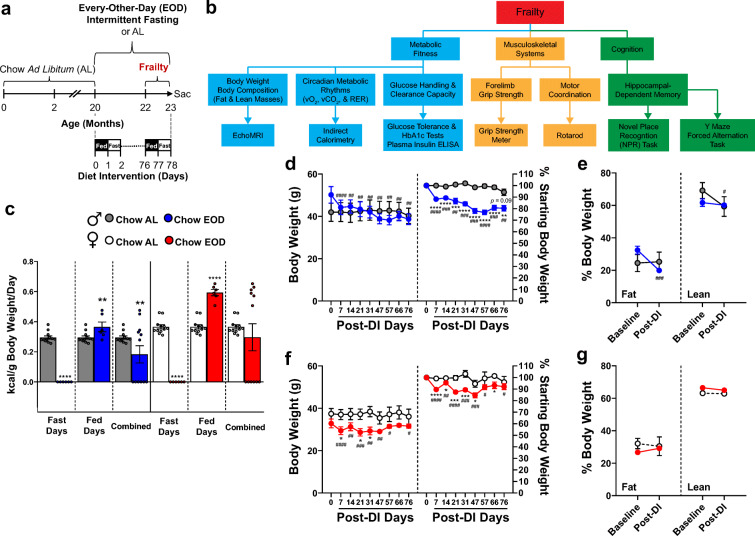
Late-life initiated EOD fasting decreases food consumption and body weight and alters body composition in a sexually dimorphic manner. **a** Graphical presentation of the experimental timeline. Twenty-month-old male and female C57BL/6 mice were placed on standard rodent chow ad libitum (Chow AL) or every-other-day intermittent fasting (Chow EOD) for 2.5 months. Frailty was determined using physiological and behavioral tests. **b** List of frailty assessment tools grouped into metabolic fitness, musculoskeletal systems, and cognition. **c** Food intake (kcal/g of body weight/day) during the fast days, fed days, and combined over the 12 time points in the male and female Chow AL and Chow EOD groups (*n* = 5–7 mice per sex per group). **d–g** Body mass and body composition in male and female mice on Chow AL and Chow EOD feeding. Absolute body weight (*left*) and % body weight compared with baseline (*right*) in the male (**d**) and female (**f**) Chow AL and Chow EOD groups from baseline to post-dietary intervention (post-DI). **e**, **g** % fat mass adjusted to body weight (*left*) and % lean mass adjusted to body weight (*right*) in the male (**e**) and female (**g**) Chow AL and Chow EOD groups at baseline and post-DI. The figures (**c–g**) depict the mean with error bars (± SEM). The asterisks indicate the significant difference between the same-sex Chow AL and Chow EOD groups. *p < 0.05, **p < 0.01, ***p < 0.001, and ****p < 0.0001. The pound signs indicate the significant within group difference between the baseline and a post-DI time point. ^#^*p* < 0.05, ^##^*p* < 0.01, ^###^*p* < 0.001, and ^####^*p* < 0.0001. See also Supplemental Figure 1

While many studies are examining the impact of EOD fasting in rodents, few have specifically delved into its late-life application (> 80 weeks of age for mice) as a means to improve sex-specific multicomponent frailty, including enhancements in hippocampal-dependent memory, as well as simultaneously investigating possible molecular mechanisms of action. Here, we report for the first time that late-life initiated EOD fasting for 2.5 months in ~80-week-old male and female C57BL/6 mice (Fig. 1a) improved multiple components of frailty (Fig. 1b), including hippocampal-dependent memory in males, but only somewhat in females. Furthermore, improved frailty was positively correlated in multiple components with augmented renal hydrogen sulfide (H_2_S) production capacity, which has been suggested as an anti-aging biomarker [77–80]. Therefore, late-life initiated EOD fasting is sufficient to reduce aging-related frailty, at least in males, and suggests that renal H_2_S production capacity may modulate the systemic effects of late-life EOD fasting on frailty.

## Materials and methods

### Animal husbandry and diet intervention

All experiments adhered to the National Institutes of Health Guide for the Care and Use of Laboratory Animals and were approved by the Cleveland Clinic Institutional Animal Care and Use Committee (IACUC), protocol numbers 2016-1778 and 2019-2258. For aged mice, male and female C57BL/6 mice were obtained between 60 and 65 weeks of age (Stock No.: 000664, Jackson Laboratories, Bar Harbor, ME) and group-housed (3–5 same-sex mice per cage) in the Cleveland Clinic Lerner Research Institute Biological Resource Unit on a 14-hr light/10-hr dark cycle, temperature between 20 and 23 °C, 30–70% relative humidity. The mice had ad libitum (AL) access to standard rodent chow (18.6% protein, 44.2% carbohydrate, and 6.2% fat; Teklad Global Rodent Diet #2918, Envigo, Madison, WI). At approximately 20 months of age, male and female cages were randomly assigned to either EOD fasting regimen (Chow EOD group: *n* = 6 males, *n* = 7 females to start) or AL access to the standard rodent chow (Chow AL group: *n* = 7 males; *n* = 6 females to start). The EOD fasting regimen consisted of repeated cycles of 24-hr consecutive removal of food access with water available AL (fast day) followed by 24-hr access to food and water AL (fed day). To circumvent possible disturbances in circadian rhythms and feeding patterns in the Chow EOD group [81, 82], the food was provided to or removed from the Chow EOD group 2–3 hrs before the dark cycle onset (19:00 hr). The daily food intake was periodically measured via manual weighing on a per cage basis over the 78-day post-dietary intervention (DI) from approximately 80 weeks of age (baseline; day 0) (see Fig. 1a for diet logistics). Similarly, 6-month-old male and female C57BL/6 mice born in the Cleveland Clinic Lerner Research Institute Biological Resource Unit were maintained on the standard rodent chow until being randomly divided into Chow AL (*n* = 3 mice/sex) and Chow EOD (*n* = 3 mice/sex) diet groups for 1 week to monitor weight and body composition changes as the young adult group.

### Frailty measures (see Fig. 1b for the list of tools)

#### Metabolic parameters

Body weight was monitored at baseline, days 7, 14, 21, 31, 45, 57, 66, and 76 post-DI for the aged group, and at baseline, day 3, and day 7 post-DI in the young group via manual weighing on a digital scale. Body composition (% fat mass and % lean mass) was assessed at baseline and at the conclusion of the study (day 78 post-DI for the aged group and day 7 post-DI for the young group) using EchoMRI body composition analysis (EchoMRI, Houston, TX). Glucose tolerance test (GTT) was performed at 13:00 hr on day 70 after a 4-hr morning fast. The mice were given an intraperitoneal glucose injection (2 g of glucose/kg body weight) followed by blood glucose measurements at time points between 0 and 120 min post-injection with an Accu-Chek glucose meter (Roche Diabetes Care, Inc., Indianapolis, IN). This procedure was repeated on day 71 to account for the differences between the fast and fed days to avoid making a type I error or have a single overnight fast influencing the result. The presented data reflect the average blood glucose levels across the two days. Hemoglobin A1c (HbA1c) levels were tested using an A1CNow blood monitor (PTS Diagnostics, Indianapolis, IN) after a 4-hr morning fast on day 78 post-DI. Plasma insulin levels were measured via the enzyme-linked immunosorbent assay (ELISA) kit (Cat. # EZRMI-13K, EMD Millipore, Billerica, MA), and plasma creatinine concentration level was measured via a creatinine colorimetric assay kit (Cat. # MAK080, Sigma-Aldrich, St. Louis, MO) per manufactures’ instructions.

#### Indirect calorimetry

Circadian metabolic rhythms were measured by the Oxymax-CLAMS indirect calorimetry (Columbus Instruments, Columbus, OH) at baseline and ~day 38 post-DI. In this system, mice were individually housed for 4-consecutive 24-hr periods to measure food intake, ambulatory activity levels, O_2_ consumption (vO_2_), CO_2_ production (vCO_2_), energy expenditure (heat; 3.815 + 1.232 × respiratory exchange ratio [RER]), and RER (vCO_2_/vO_2_), which reflects the type of substrate that is being utilized. Double-plotted actograms were composed using the averaged data for each time point separated by the fed and fast days with a time of the day expressed in Zeitgeber time (ZT). Similar to as when in the standard animal cages and vivarium housing rooms, Chow EOD mice were fed or fasted at approximately ZT9 daily. To observe changes in the circadian rhythm, the amplitudes/fluctuations of the rhythm were determined using the area under the curve (AUC) method. In addition, to test whether circadian pattern (i.e., timing of a reference point in the cycle) was shifted, the average dark phase AUC and light phase AUC were compared.

#### Behavioral tasks

To reduce the effects of experimenter-induced anxiety and stress on behavior-related assays, mice were handled three consecutive days (2-min per mouse on the 1st day, 1-min each on the 2nd+ days) at least 48 hr before the behavioral assays. All behavioral tasks were carried out during the light cycle at the Rodent Behavior Core testing suite in the Cleveland Clinic Lerner Research Institute Biological Resource Unit. The battery of behavioral tasks was conducted in the order of Y maze forced alternation task, forelimb grip strength, open field, novel place recognition task, and rotarod, and the order was kept constant for each animal. The mice were habituated in the testing suite at least 30 min before and after each behavioral testing day. They were given at least 48 hr apart in-between each task to minimize any carry-over effects. Males and females were tested separately in a random order for each task. Behavioral apparatuses were wiped with 70% ethanol in-between trials and mice to eliminate olfactory cues. Behavioral sessions were digitally recorded using a CCTV camera mounted to the ceiling positioned directly above behavioral apparatuses. All recorded behavioral sessions were analyzed using the behavior analysis software TopScanLite Version 2.0 (Clever Sys Inc., Reston, VA) or EthoVision XT 13.0 (Noldus, Wageningen, the Netherlands). Outliers from each task were excluded from further analyses.

#### Y maze forced alternation task

Short-term memory was tested using the Y maze forced alternation task at baseline and ~day 50 posy-DI. This task requires the functional integrity of the hippocampus [83, 84]. Mice were trained in a Y maze (each arm 12 cm H, 38.5 cm D, 9 cm W) surrounded by intra and extra maze visual cues. During the acquisition trial, mice were released into a distal end of one of two randomly selected starting arms facing away from the center of the Y maze. The mice did not have access to the third arm, also termed the target arm, during training. Mice were allowed to freely investigate the two arms for 15 min. Then they were placed back into their home cages in the behavioral testing suite. After a 2-hr inter-trial interval (ITI), a 4-min memory probe test was given. The mice were released into the Y maze in an identical manner as training, but the dividing wall to the target arm was removed. They were allowed to freely investigate the arms. The duration and number of entries to each arm and velocity/speed during the retention trial were recorded for further analyses. Two-hour ITI was chosen given that 2 hr after training, young healthy rats enter the target arm and spend more time above chance (33%) during the retention trial [85]; however, aged mice fail to show a preference for the target arm with this ITI duration [86–88]. This task was repeated on ~day 50 with different intra and extra maze cues.

#### Open field

During the habituation phase of the novel place recognition test, the mice were given a 10-min open field trial at baseline and ~day 59 post-DI. An open field (40.5 cm H × 51.0 cm D × 61.0 cm W) was virtually divided into 3 arenas: outer, middle, and center. Behavior was digitally recorded for further analysis. The number of entries, duration, and latency to the center arena (anxiety-like behavior), rearing (exploratory/anxiolytic behavior), and distance traveled/path length and velocity (locomotion) were measured.

#### Novel place recognition test

Twenty-four hours after the open field test, object location memory was tested using the novel place recognition (NPR) test at baseline and ~day 60 post-DI. In this task, two identical objects (random assignment to either glass media bottles filled with corncob bedding or plastic sippy cups filled with water) were placed at fixed and equidistant locations in an open field. Mice were released into the outer edge of the open field facing away from the center of the arena and were allowed to freely investigate the objects and the open field for 5 min. Then they were placed back into their home cage and were remained in the behavioral testing suite for a minimum of 30 min before transported back to the vivarium. After a 24 hr ITI, a 2-min memory probe test was given. Mice were reintroduced to the identical environment and objects, but one of the objects was moved to a new location, located at an opposite corner relative to the original location. The other object remained at the same location. Mice were allowed to freely investigate in the open field. Object investigation duration and proximity to the objects during the retention trial were recorded for further analyses. A discrimination index was calculated as the duration of object investigation at a new location minus the duration of investigation of an object that has not moved, divided by the total object investigation duration. Thus, a positive score on the discrimination index means the mouse investigated the object at a new location longer. Due to the innate preference of rodents for novelty, rodents with intact hippocampal function will investigate an object moved to a new location more than an unmoved object [89–93]. Also, proximity to the objects was calculated as the ratio of the distances between the center of the moved object and the tip of the mouse nose divided by the distance between the center of the unmoved object and the tip of the mouse nose. Thus, a greater proximity ratio indicates that the mouse directed its face closer to the unmoved object than to the moved object. Twenty-four-hour ITI was chosen given that 24 hr after training, young 2–4-month-old mice show a preference for a moved object; however, 22–24-month-old mice do not [94]. This task was repeated on ~day 60 post-DI with different objects and placements.

#### Muscular strength and neuromuscular function parameters

Forelimb grip strength was assessed at baseline and ~day 48 using a grip strength meter (Columbus Instruments, Columbus, OH). Each mouse was given five trials, and average grip strength (g) and average grip strength adjusted to body weight (grip strength [g]/body weight [g]) were recorded. The fold change in strength was then calculated. Motor coordination was tested using a rotarod (Columbus Instruments, Columbus, OH). Briefly, following the acclimation trial (4 rpm for 30 sec), mice were given a total of four trials (max duration: 300 sec, max speed: 50 rpm, acceleration: 1 rpm inclement per 11 sec per trial) with 5-min ITI. Latency and speed at which the mouse fell from or passively rotated on a revolving rod were recorded in trial 2–4 as a measure of motor coordination.

### Molecular and hormonal parameters

#### Filter paper-embedded lead acetate endpoint assay

The endogenous H_2_S production capacities of liver, kidney, muscle (quadriceps), heart, and brain were measured by the lead sulfide method as previously described [95, 96]. Briefly, tissues were immediately harvested from euthanized animals and flash-frozen. Flash-frozen tissues were then homogenized and lysed in freshly prepared 1× passive lysis buffer (Cat. # E1941, Promega, Madison, WI). One hundred micrograms of normalized protein with the 150 μL of reaction mixture containing 10 mM L-cysteine (Cat. #. 168149, Sigma-Aldrich, St. Louis, MO) and 1 mM pyridoxal phosphate (Cat. # 9255, Sigma-Aldrich, St. Louis, MO) in phosphate-buffered saline was placed in 96-well plates. A lead acetate-embedded H_2_S detection filter paper was placed on top of the well plates and incubated at 37 °C for 2–24 hr. H_2_S production capacity was quantified by measuring the lead sulfide darkening/density of the paper using the IntDen function in ImageJ (Rasband, W.S., ImageJ, U. S. National Institutes of Health, Bethesda, MD, USA, https://imagej.nih.gov/ij/, 1997–2018).

#### Real-time quantitative PCR analysis

Gene expression of (1) neuroinflammatory cytokine nuclear factor kappa B (NF-κB) and its downstream pro-inflammatory cytokines interleukin 1 beta (IL1β), interleukin 6 (IL6), and tumor necrosis factor-alpha (TNF-α), (2) a neuroprotective chaperone protein PARK7/DJ-1, (3) anorexigenic and orexigenic genes proopiomelanocortin (POMC), and agouti-related protein (AgRP), and (4) the brain-derived neurotrophic factor (BDNF) gene in the hypothalamus was quantified using a real-time quantitative PCR (qPCR) method as previously described [97] with slight modifications per manufactures’ instructions. Briefly, total RNA was isolated with TRIzol reagent (Cat. # 15596026, Invitrogen, Waltham, MA), and single-stranded cDNA was synthesized using Verso cDNA kit (Cat. # AB1453A, Thermo Scientific, Waltham, MA). qPCR was performed with Fast SYBR Green Master Mix (Cat. # 4385612, Applied Biosystems, Waltham, MA) with the primer mix containing forward and reverse ReadyMade Primers (Integrated DNA Technologies, Inc., Research Triangle Park, NC) of a targeted gene. Plates were run on Applied Biosystems StepOnePlus Real-Time PCR System. Targeted gene expression was measured using a relative quantitation (ΔΔC_T_) method comparing against the reference/housekeeping gene β-actin. Fold changes were normalized to the Chow AL group. Primers used were the following: NF-κB F: GGAGACTCGTTCCTGCACTTGG, NF-κB R: AACAAGAGCGAAACCAGGTCAGG, IL6 F: TACCACTTCACAAGTCGGAGGC, IL6 R: CTGCAAGTGCATCATCGTTGTTC, IL1β F: TGGACCTTCCAGGATGAGGACA, IL1β R: GTTCATCTCGGAGCCTGTAGTG, TNF-α F: CATCTTCTCAAAATTCGAGTGACAA, TNF-α R: TGGGAGTAGACAAGGTACAACCC, DJ-1 F: GCAGGAAGGGCCTCATAGCT, DJ-1 R TGTTGTGACCTTGCATCCAAA, POMC F: AAGAGCAGTGACTAAGAGAGGCCA, POMC R: ACATCTATGGAGGTCTGAAGCAGG, AgRP F: AGGGCA TCAGAAGGCCTGACCA, AgRP R: CTTGAAGAAGCGGCAGTAGCAC, BDNF F: GGCTGACACTTTTGAGCACGT, BDNF R: CTCCAAAGGCACTTGACTGCTG, β-actin F: AGGCTGTGCTGTCCCTGTATG, and β-actin R: ACCCAAGAAGGAAGGCTGGAAA.

### Statistical analyses

Figures depict the means ± SEM with *n* of 3–7 per group as indicated in the figure legends. The behavior analysis software TopScanLite Version 2.0 (Clever Sys Inc., Reston, VA) or EthoVision XT 13.0 (Noldus, Wageningen, the Netherlands) was used for the appropriate behavioral tasks. All data were analyzed using IBM SPSS Statistics for Windows, Version 25.0 (IBM Corporation, Armonk, NY) or GraphPad Prism for Windows, Version 8.0 (GraphPad Software, Inc., La Jolla, CA). Results were considered statistically significant when *p* values were less than 0.05.

For food intake analysis, a 2 × 2 × 3 analysis of variance (ANOVA) was used to test the effects of sex (male vs. female), DI (Chow AL vs. Chow EOD), and day (fast days, fed days, and combined). Post hoc comparisons were performed using Tukey’s HSD or independent samples *t* tests. Due to the sexually dimorphic effect of EOD fasting on food intake, for other dependent variables, independent samples *t* tests were carried out to test the effects of DI, and dependent samples *t* tests were conducted to test the effects of time (baseline vs. the various post-DI time points and the light vs. the dark cycle) separately for each sex. For the forced alternation task, one-sample *t* tests were conducted to see whether each of the DI group spent in the target arm below chance (hypothetical value of 80 sec). The Pearson product-moment correlation matrices (one-tailed) were computed to test the a priori-predicted relationships (1) among the frailty measures in both males and females, (2) between renal H_2_S production and frailty measures in males, and (3) between renal H_2_S production and hypothalamic gene expression in males.

## Results

### Late-life initiated EOD fasting decreases food consumption and body weight and alters body composition in a sexually dimorphic manner

To test the effects of EOD fasting on caloric intake in aged mice, food consumption per BW was measured periodically over the 78 days of DI to determine average food intake during the fast days, the fed days, and combined (Fig. 1c). There were significant main effects of sex (*p* < 0.0001), DI (*p* < 0.0001), and food intake day (*p* < 0.0001) and significant interaction effects of sex × food intake day (*p* < 0.05), DI × food intake day (*p* < 0.0001), and sex × DI × food intake day (*p* < 0.05) on the kcal of food consumed. Specifically, females consumed more food per BW relative to males. As expected, mice that remained on the AL feeding regimen (Chow AL) consumed more food overall than the mice placed on the EOD fasting regimen (Chow EOD). Specifically, the male Chow EOD group consumed more food during the fed days (*M* = 0.37 kcal/g BW/day) by 124% relative to the male Chow AL group (*M* = 0.30 kcal/g BW/day). Therefore, the male Chow EOD group consumed less food per BW on average (*M* = 0.18 kcal/g BW/day), specifically by − 38%, relative to the male Chow AL group (Fig. 1c). Similarly, the female Chow EOD group consumed more food per BW during the fed days (*M* = 0.59 kcal/g BW/day) relative to the female Chow AL group (*M* = 0.37 kcal/g BW/day), specifically by 162%. As a result, overall food intake was not significantly different between the female Chow AL and the Chow EOD groups (*M* = 0.30 kcal/ g BW/day; − 19% change; Fig. 1c). Thus, while EOD fasting was an effective caloric restricting regimen in aged male mice, it failed to decrease overall food intake in aged female mice. Given that the DI-induced food/caloric intake differed across the male and female aged mice, subsequent behavioral and physiological measures were analyzed separately for each sex.

Body weight and body composition in the form of fat and lean masses were recorded periodically from baseline to day 76 post-DI for aged mice (Fig. 1d–g) and from baseline to day 7 post-DI for the young adult mice cohort (Supplemental Figure 1A–D). EOD fasting in males reduced their BW continuously during the entire span of DI, as their BW was decreased relative to baseline on each BW recording day (Fig. 1d and Supplemental Figure 1A). Body composition analysis by EchoMRI [98, 99] revealed the decrease in BW was mainly due to a decrease in % fat mass relative to baseline (Fig. 1e and Supplemental Figure 1B). The aged male Chow AL group maintained their BW during the entire span of the experiment, except for trend (*p* = 0.089) in decreased mass in their final BW on day 76 relative to baseline (Fig. 1d). This subtle decrease was most likely due to a decrease in their % lean mass, with their % fat mass unaffected (Fig. 1e). EOD fasting in females decreased their BW soon after the initiation of the DI (Fig. 1f and Supplemental Figure 1C). However, in contrast to males, BW-reducing effects of EOD fasting diminished before the end of the experiment. Specifically, BWs of the female Chow EOD group were continuously decreased relative to the female Chow AL group until day 47, but not beyond this time point (Fig. 1f). Similarly, in the young adult female group, weight loss was more dramatic at 3 days rather than 7 days post-DI (Supplemental Figure 1C). Body composition analysis revealed % fat mass and % lean masses were not affected by DI or by time in aged females (Fig. 1g). Likewise, young females had a less dramatic change in body mass composition compared to young males under EOD fasting (Supplemental Figure 1D). Thus, EOD fasting decreased BW primarily by decreasing fat mass in aged males, but not in aged females, with analogous results observed in young adult mice.

### Late-life EOD fasting attenuates metabolic inflexibility

**Fig. 2:**
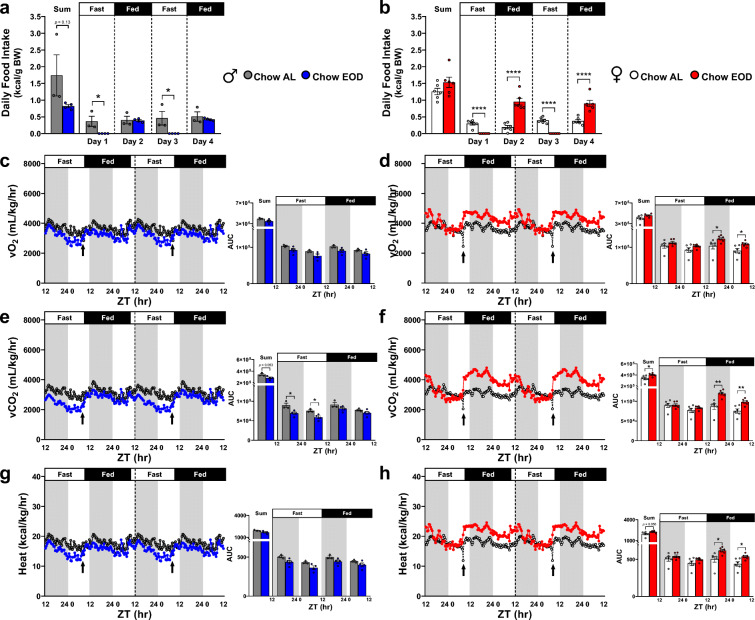
Late-life EOD fasting affects body weight-adjusted resting metabolism. **a**, **b** Average food intake (kcal/g of body weight) per mouse over the 4-day period in the metabolic chamber was determined by manual weighing daily in the male (**a**) and female (**b**) Chow AL and Chow EOD groups (*n* = 3–6 mice per sex per group). **c–h** The body weight-adjusted average vO_2_ (mL/kg/hr) (**c**, **d**), vCO_2_ (mL/kg/hr) (**e**, **f**), and heat (kcal/kg/hr) (**g**, **h**) were measured approximately every 20 min. The figures (**c**–**h**) depict the mean with no error bars for a representation purpose, with insets providing the area under the curve (AUC) of combined/summation of area, fast day 12:12 light:dark cycle, and fed day 12:12 light:dark cycle for each measure. The inset figures depict the mean with error bars (± SEM). The asterisks indicate the significant difference between the same-sex Chow AL and Chow EOD groups. *p < 0.05, **p < 0.01, and ****p < 0.0001. See also Supplemental Figure 2

As metabolic flexibility declines with age [100, 101], we next tested resting whole body metabolic activity using indirect calorimetry in a 12-chamber open-circuit Oxymax Comprehensive Lab Animal Monitoring System (CLAMS; Columbus Instruments, Columbus, OH). Individual food intake patterns for both males and females as determined by manual weighing daily (Fig. 2a, b) were similar to the entire experimental span of experimenter manually determined food intake (Fig. 1c), and the time when the mice initiated major feeding was similar between Chow AL and Chow EOD groups (Supplemental Figure 2A, B). Specifically, in males, there was a non-significant decreasing trend (*p* = 0.13) in overall kcal consumption of food in the Chow EOD group relative to the Chow AL group (Fig. 2a). However, in females, overall kcal consumption was not affected by the EOD fasting due to an increase in food consumption during the fed days relative to the female Chow AL group (Fig. 2b).

To determine the contributions of the whole body (i.e., various organs with adipose and lean tissues) to metabolism, we calculated vO_2_, vCO_2_, and heat by adjusting to total BW. The significant circadian rhythms were observed on metabolic processes in all of the groups, as vO_2_, vCO_2,_ and heat during the dark phases were elevated relative to the light phases (all *p* < 0.05, except for vCO_2_ in the male Chow AL group *p* = 0.054; Table 1). In comparison with the male Chow AL group, EOD fasting in males did not affect overall vO_2_, as well as segmented vO_2_, which were separated by the light and dark phases and by fast and fed days (Fig. 2c). However, in females, EOD fasting increased the amplitudes of vO_2_ during the dark and light phases of the fed days relative to the Chow AL group, which became apparent at the onset of feeding (≈ ZT9, indicated by the arrow) while the food was present (Fig. 2d). Overall vCO_2_ was slightly, but non-significantly, decreased (*p* = 0.053) in the male Chow EOD group relative to the Chow AL group, which was driven by the decrease in vCO_2_ during the dark and light phases of the fast days (Fig. 2e). In contrast, EOD fasting in females increased the amplitude of vCO_2_ during the dark and light phases of the fed days relative to the female Chow AL group, which contributed to an increased overall vCO_2_ (Fig. 2f). Furthermore, EOD fasting in males did not affect heat/energy expenditure (Fig. 2g). However, EOD fasting in females tended to increase overall heat production (*p* = 0.056), which was due to an increase in the amplitude of heat during the dark and light phases of the fed days (Fig. 2h). Importantly, circadian rhythms in regard to feeding timing and movement activity patterns were minimally affected by the EOD fasting, most likely due to strict adherence to re-feeding at the same time the Chow AL group mice naturally consume their food, i.e., around the start of the dark phase (Table 1, Supplemental Figure 2A–F). Taken together, these findings indicate EOD fasting enhanced whole body-generated metabolic flexibilities and capabilities in a sex-dependent manner.

**Table 1:** Late-life EOD fasting selectively attenuates aging-related circadian pattern disruptions. The dependent samples *t* test summary table for the area under the curve comparisons on each metabolic measures between the Chow AL and Chow EOD groups separated by sex (*n* = 3–6 mice per sex per group) and parameters normalized to whole body weight (*top*), lean tissue (*middle*), or non-applicable mass normalization (*bottom*). The *p* values < 0.05 indicate the significant difference between the same-sex Chow AL and Chow EOD groups

				Dark Phase Mean AUC	Light Phase Mean AUC	M AUC Diff.	SD	SEM	df	*t*	*p*
Whole Body (Adipose & Lean Tissues)	Male	vO_2_	Chow AL	205164.67	180632.33	24532.33	816.24	471.25	2.00	52.06	0.000
			Chow EOD	181691.50	158566.75	23124.75	4920.24	2460.12	9.40	3.00	0.003
		vCO_2_	Chow AL	180204.67	153042.67	27162.00	11376.09	6567.99	2.00	4.14	0.054
			Chow EOD	149739.00	128654.25	21084.75	4883.85	2441.93	8.63	3.00	0.003
		Heat	Chow AL	1004.67	877.63	127.03	12.31	7.11	2.00	17.87	0.003
			Chow EOD	877.65	763.43	114.22	24.72	12.36	9.24	3.00	0.003
	Female	vO_2_	Chow AL	206735.67	182511.33	24224.33	13036.52	5322.14	5.00	4.55	0.006
			Chow EOD	236085.33	211639.50	24445.83	8917.85	3640.70	6.72	5.00	0.001
		vCO_2_	Chow AL	176418.00	152313.67	24104.33	10630.56	4339.91	5.00	5.55	0.003
			Chow EOD	209936.83	181890.83	28046.00	9651.99	3940.41	7.12	5.00	0.001
		Heat	Chow AL	1006.07	883.90	122.17	62.36	25.46	5.00	4.80	0.005
			Chow EOD	1158.70	1031.35	127.35	45.40	18.53	6.87	5.00	0.001
Lean Tissue	Male	vO_2_	Chow AL	271010.33	238528.00	32482.33	3348.20	1933.08	2.00	16.80	0.004
			Chow EOD	279753.25	244150.00	35603.25	7290.20	3645.10	9.77	3.00	0.002
		vCO_2_	Chow AL	237096.33	201956.67	35139.67	11458.28	6615.44	2.00	5.31	0.034
			Chow EOD	230542.50	198099.00	32443.50	7130.87	3565.43	9.10	3.00	0.003
		Heat	Chow AL	1325.93	1158.83	167.10	5.12	2.95	2.00	56.56	0.000
			Chow EOD	1351.38	1175.50	175.88	36.64	18.32	9.60	3.00	0.002
	Female	vO_2_	Chow AL	348637.33	307639.33	40998.00	22113.06	9027.62	5.00	4.54	0.006
			Chow EOD	357972.50	321310.83	36661.67	12207.19	4983.57	7.36	5.00	0.001
		vCO_2_	Chow AL	297707.50	256769.17	40938.33	18806.71	7677.81	5.00	5.33	0.003
			Chow EOD	318298.00	276180.50	42117.50	13119.99	5356.22	7.86	5.00	0.001
		Heat	Chow AL	1696.97	1489.92	207.05	106.78	43.59	5.00	4.75	0.005
			Chow EOD	1757.83	1566.12	191.72	62.08	25.35	7.56	5.00	0.001
	Male	RER	Chow AL	47.36	44.03	3.33	2.92	1.68	2.00	1.98	0.187
			Chow EOD	44.48	42.02	2.46	0.33	0.16	14.92	3.00	0.001
		Food Int.	Chow AL	6.75	4.77	1.98	1.99	1.40	1.00	1.41	0.393
			Chow EOD	3.50	2.33	1.17	0.72	0.41	2.84	2.00	0.105
		Crossing	Chow AL	31802.00	12618.00	19184.00	17330.79	10005.93	2.00	1.92	0.195
			Chow EOD	45679.00	20148.75	25530.25	10538.73	5269.37	4.85	3.00	0.017
		Rearing	Chow AL	23635.00	9291.00	14344.00	8968.47	5177.95	2.00	2.77	0.109
			Chow EOD	51692.00	16853.25	34838.75	39075.63	19537.82	1.78	3.00	0.173
	Female	RER	Chow AL	46.03	43.35	2.68	0.69	0.28	5.00	9.48	0.000
			Chow EOD	47.59	44.40	3.19	0.69	0.28	11.25	5.00	0.000
		Food Int.	Chow AL	12.16	3.06	9.10	12.77	6.39	3.00	1.43	0.249
			Chow EOD	4.30	3.21	1.09	0.80	0.36	3.04	4.00	0.038
		Crossing	Chow AL	32990.83	13282.00	19708.83	11632.89	4749.11	5.00	4.15	0.009
			Chow EOD	46478.50	21035.83	25442.67	11076.77	4522.07	5.63	5.00	0.002
		Rearing	Chow AL	18925.83	8252.83	10673.00	7407.37	3024.05	5.00	3.53	0.017
			Chow EOD	22080.50	9577.67	12502.83	6221.64	2539.97	4.92	5.00	0.004

**Fig. 3:**
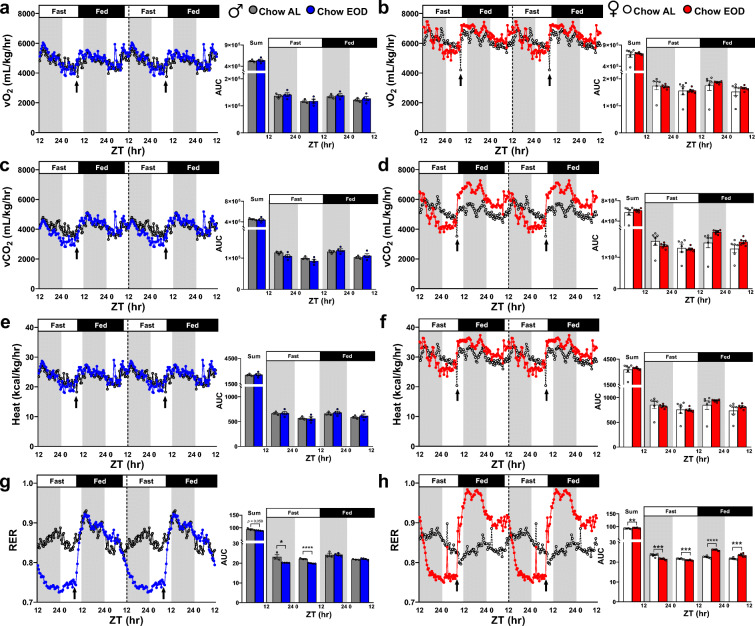
Late-life EOD fasting affects lean mass-adjusted resting metabolism. **a**–**h** The lean mass-adjusted average vO_2_ (mL/kg/hr), vCO_2_ (mL/kg/hr), and heat (kcal/kg/hr), as well as RER, were measured approximately every 20 min over the 4-day period in the metabolic chamber in the male (**a, c, e, g**) and in female (**b, d, f, h**) Chow AL and Chow EOD groups (*n* = 3–6 mice per sex per group). The figures (**a**–**h**) depict the mean with no error bars for a representation purpose, while the insets show the AUC of combined/summation of area, fast day 12:12 light:dark cycle, and fed day 12:12 light:dark cycle for each measure. The inset figures depict the mean with error bars (± SEM). The asterisks indicate the significant difference between the same-sex Chow AL and Chow EOD groups. *p < 0.05, **p < 0.01, ***p < 0.001, and ****p < 0.0001

Given that (1) loss or gain of adipose and lean masses are not in a reciprocal relationship (Fig. 1e, g and 2) lean tissue is more metabolically active than fat tissue [102–105], we next re-examined our indirect calorimetry data by adjusting vO_2_, vCO_2_, and heat to the individual lean mass of each mouse (Fig. 3). In both males and females, EOD fasting did not affect the overall degree of circadian rhythmicity (Table 1), vO_2_ (Fig. 3a, b), vCO_2_ (Fig. 3c, d), and heat (Fig. 3e, f) across the dark and light phases or the fast and fed days.

Remarkably, the effects of EOD fasting became prominent on the RER calculated by dividing the vCO_2_ by the vO_2_ (Fig. 3g, h). Specifically, EOD fasting produced a dynamic circadian rhythm of RER by extending the period (i.e., the time interval between peaks [106]) in males (Fig. 3g) and females (Fig. 3h). In particular, this effect was more pronounced in females, given that EOD fasting increased the amplitudes of RER during the dark and light phases of the fed days in females relative to the Chow AL group, but not in males. These findings reflect that EOD fasting resulted in a larger shift from fatty acid to carbohydrate oxidation depending on food availability, which was greater in females than males. In sum, these findings indicate that EOD fasting initiated late in life is effective at inducing differential macronutrient oxidation, with the extent of net RER amplitude changes being sexually dimorphic.

### Late-life EOD fasting improves glucose handling and clearance capacity

**Fig. 4:**
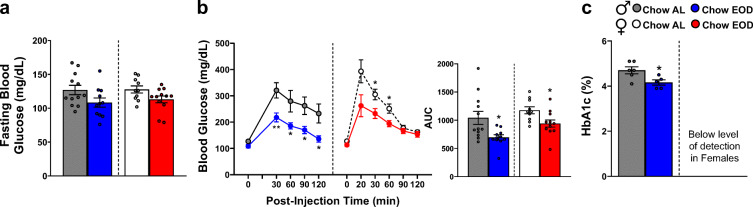
Late-life EOD fasting improves glucose handling and clearance capacity. **a** Average 4–6-h fasting blood glucose levels (mg/dL) prior to the start of the glucose tolerance test (GTT) taken on adjacent fasted and fed days in male (*left*) and female (*right*) Chow AL and Chow EOD groups (*n* = 5–6 mice per sex per group per day). **b** Average blood glucose levels (mg/dL) at time points between 0 and 120 min following an intraperitoneal injection of glucose (2 g of glucose/kg of body weight) from the GTT being performed on adjacent fasted and fed days in the male (*left*) and female (*right*) Chow AL and Chow EOD groups (*n* = 5–6 mice per sex per group per day). The inset shows the AUC of 0–120 min post-injection blood glucose levels in the male (*left*) and female (*right*) Chow AL and Chow EOD groups. **b** HbA1c levels in the male Chow AL (*n* = 6) and Chow EOD groups (*n* = 5). The figures (**a**, **b**) depict the mean with error bars (± SEM). The asterisks indicate the significant difference between the same-sex Chow AL and Chow EOD groups. *p < 0.05 and **p < 0.01. See also Supplemental Figure 4

Given that EOD fasting enhanced RER amplitudes indicative of differential energy source utilization, we next tested glucose handling and clearance capacity using the glucose tolerance test (GTT). Figure 4a depicts 4–6-h fasting blood glucose levels at baseline prior to glucose administration averaged from adjacent fed and fasted days, while Fig. 4b shows each time point prior to and post glucose administration compiled and averaged from the GTT performed on adjacent fasted and fed days. In males and females, the Chow EOD groups did not have a marked difference in baseline blood glucose levels compared to the Chow AL groups (Fig. 4a). However, both male and female Chow EOD groups showed lower blood glucose levels, enhanced glucose tolerance, and handling capacity compared to the AL control groups post glucose administration (Fig. 4b). Specifically, there were − 33% and − 20% changes in the area under the curve (AUC) for the male and female Chow EOD groups relative to controls, respectively (Fig. 4b). Additionally, improved glucose handling in both male and female EOD groups was most prominent at the 20–30-min post glucose injection (Fig. 4b). However, only male Chow EOD mice, but not female Chow EOD mice, maintained lower blood glucose levels at later 90–120-min time points compared to Chow AL mice. This can be attributed to female Chow AL mice obtaining lower blood glucose levels similar to their EOD counterparts at these later time points in the GTT (Fig. 4b), suggesting female mice may inherently have better glucose tolerance than males at advanced ages. Likewise, Hemoglobin A1C, a glycated form of hemoglobin and a stable clinical blood marker of diabetes [107, 108], was decreased in the male Chow EOD group compared to the Chow AL group (Fig. 4c), but was not detectable in female mice (Fig. 4c). These positive effects of EOD fasting on glucose tolerance and handling were independent of any changes in circulating insulin levels (Supplemental Figure 4).

### Late-life EOD fasting enhances musculoskeletal systems

**Fig. 5:**
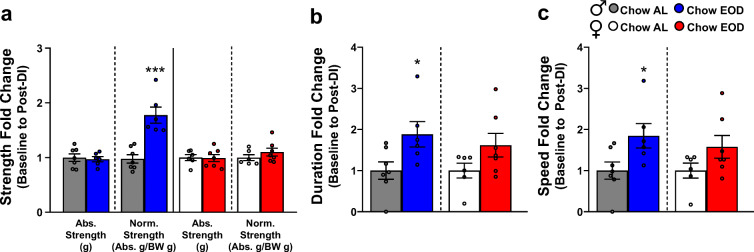
Late-life EOD fasting enhances musculoskeletal systems. **a** Baseline to post-DI fold change of average forelimb grip strength (abs. strength [g]) and body weight-adjust grip strength (abs. strength [g]/body weight [g]) in the male (*left*) and female (*right*) Chow AL and Chow EOD groups (*n* = 6–7 mice per sex per group). **b**, **c** Baseline to post-DI fold change of rotarod duration/latency to fall in the male (**b** *left*) and female (**b** *right*) Chow AL and Chow EOD groups (*n* = 6–7 mice per sex per group). Baseline to post-DI fold change of the average max speed in the male (**c** *left*) and female (**c** *right*) Chow AL and Chow EOD groups. The figures (**a–c**) depict the mean with error bars (± SEM). The asterisks indicate the significant difference between the same-sex Chow AL and Chow EOD groups. *p < 0.05 and ***p < 0.001

Hallmarks of aging include the decline in lean mass concurrent with muscle strength and coordination [109–112]. As we showed declines in lean mass that were somewhat prevented by EOD fasting primarily in male mice (Fig. 1e, g), we next examined the impact of late-life initiated EOD fasting on muscular strength and coordination. Forelimb muscle strength was measured using a grip strength meter at baseline and post-DI, and the aging- and diet-induced change in forelimb muscle strength calculated (Fig. 5a). In males, there was no significant difference in absolute forelimb strength before and after post-DI for either diet group. However, the enhancing effect of EOD fasting was apparent when grip strength was normalized to their body weight. Specifically, EOD fasting in male mice increased forelimb grip strength 2-fold post-DI when compared with the male Chow AL group (Fig. 5a). In contrast, there was no effect of EOD fasting on forelimb grip strength in female mice, as there was no change in their absolute forelimb grip strength nor in the normalized grip strength before and after the DI (Fig. 5a).

The rotarod test measures cerebellum-mediated [113, 114] motor coordination, motor learning, and balance in rodents [115, 116]. The average latency to fall and the speed at which mice lost their balance or passively rotated were determined at baseline and post-DI, and the fold changes were calculated. In male mice, EOD fasting improved their duration on the rotarod (Fig. 5b) and the fastest speed at which they could maintain their balance (Fig. 5c) post-DI when compared with the Chow AL group. However, these effects were not observed in the female Chow EOD group (Fig. 5b, c). Taken together, these findings suggest that EOD fasting functionally improves motor coordination and balance in males, but not in females, possibly by promoting centrally regulated motor learning during aging.

### Late-life EOD fasting enhances hippocampal-dependent short-term memory

**Fig. 6:**
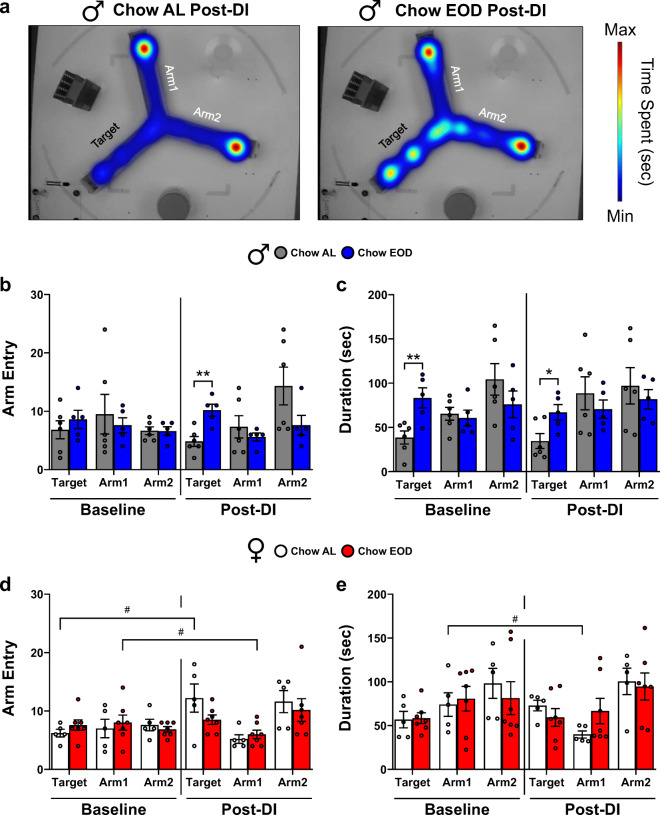
Late-life EOD fasting enhances hippocampal-dependent short-term memory. **a** Representative heatmap images of post-DI Y maze forced alternation task performance in the male Chow AL (*left*) and Chow EOD groups (*right*). **b**–**e** Number of arm entries at baseline in the male (**b** *left*) and female (**d** *left*) Chow AL and Chow EOD groups and post-DI in males (**b** *right*) and females (**d** *right*). Duration in each arm at baseline in the male (**c** *left*) and female (**e** *left*) Chow AL and Chow EOD groups and post-DI in males (**c** *right*) and females (**e** *right*). *n* = 5–7 mice per sex per group. The figures (**a**–**e**) depict the mean with error bars (± SEM). The asterisks indicate the significant difference between the same-sex Chow AL and Chow EOD groups. *p < 0.05 and **p < 0.01. The pound signs indicate the significant within group difference between the baseline and a post-DI time point. ^#^*p* < 0.05. See also Supplemental Figure 6

Spatial and episodic learning and memory decline with age [112], primarily due to the disruption of normal hippocampal activity [117]. Thus, we ran a battery of tests to determine if late-life initiated EOD fasting preserves and/or improves hippocampal function. First, male and female mice were tested in the Y maze forced alternation task at baseline and post-DI. They were given a 15-min training trial and a 4-min retention trial 2 hr later, with a representative data output shown in Fig. 6a. Young adult rodents have an innate curiosity toward novelty, thus in this task, they show an increased preference for the novel/target arm [85, 118]. However, as animals age, it is not well understood how this preference changes as a function of age and diet. At baseline prior to DI, there was no significant difference between the male Chow AL and the Chow EOD groups on the number of entries to the target arm (Fig. 6b). In contrast, EOD fasting in males increased the number of target arm entries compared with the male Chow AL group post-DI (Fig. 6b). This effect was not due to a difference in their moving speed (Supplemental Figure 6A). Furthermore, there was a difference between the male Chow AL and the Chow EOD groups on the duration in the target arm at baseline and post-DI (Fig. 6c). This effect was due to Chow AL group spending time in the target arm below chance (80 sec) at both time points (*M* = 38.55 sec, *SD* = 18.11, one-sample *t* test *p* = 0.002 baseline; *M* = 34.54 sec, *SD* = 20.82, one-sample *t* test *p* = 0.003 post-DI), while this was not observed in the male Chow EOD group (one-sample *t* tests *p* > 0.05 baseline and post-DI). The female Chow AL and the Chow EOD groups were not significantly different on the number of target arm entries (Fig. 6d) and the target arm duration (Fig. 6e) at both baseline and post-DI. In sum, while late-life initiated EOD fasting did not produce a robust Y maze short-term memory improvement, it did provide benefits to some extent in spatial memory for males.

### Late-life EOD fasting attenuates aging-related anxiety-like behavior and maintains motor activity

**Fig. 7:**
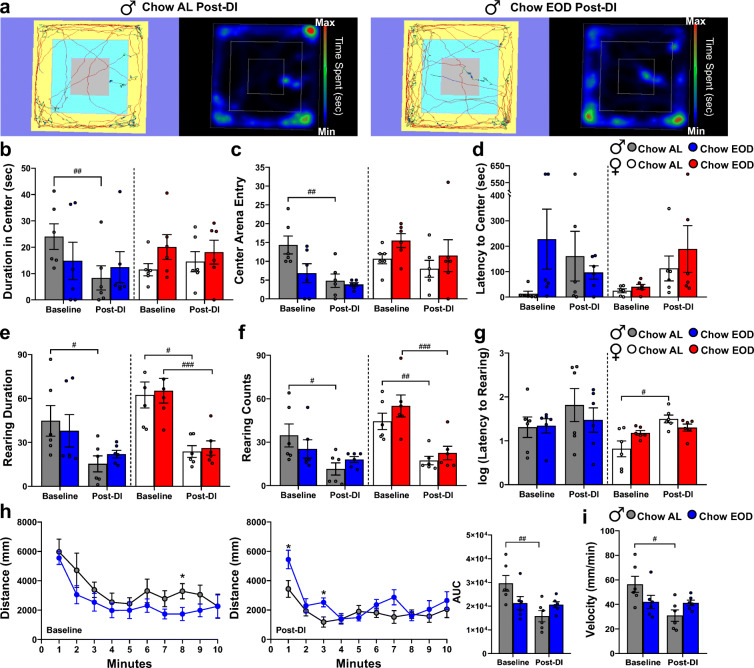
Late-life EOD fasting attenuates aging-related anxiety-like behavior and maintains motor activity in males. **a** Representative tracing images (*left*) and heatmaps (*right*) of open field performance in the male Chow AL (*left*) and Chow EOD groups (*right*). **b**–**g** Open field performance as a measure of anxiety-like behavior. Duration (**b**), number of entries (**c**), and latency to the virtual center arena (**d**) and duration (**e**), frequency (**f**), and log-transformed latency to rearing (**g**) at baseline and post-DI in the male (**b**–**g** *left*) and female (**b**–**g** *right*) Chow AL and Chow EOD groups (*n* = 6 mice per sex per group). **h**, **i** Open field performance as a measure of locomotion. Distance traveled/path length in the open field at baseline (**h** *left*) and post-DI (**h** *right*) in the male Chow AL (*n* = 6) and Chow EOD groups (*n* = 6). The AUC for distance traveled at baseline and post-DI in the male Chow AL and Chow EOD groups are shown in the inset. Velocity/speed in the open field at baseline and post-DI in the male Chow AL and Chow EOD groups (**i**). The figures (**b**–**i**) depict the mean with error bars (± SEM). The asterisks indicate the significant difference between the same-sex Chow AL and Chow EOD groups. *p < 0.05. The pound signs indicate the significant within group difference between the baseline and a post-DI time point. ^#^*p* < 0.05, ^##^*p* < 0.01, and ^###^*p* < 0.001.See also Supplemental Figure 7

The prevalence of anxiety disorder is high in older adults [119], which increases in an age-dependent manner in mice [112]. The effects of EOD fasting on anxiety-like behavior, locomotion, and rearing were measured in an open field test for 10 min, with a representative data output shown in Fig. 7a. The duration, number of entries, and latency to the virtual center arena were measured as anxiety levels. Animals with heightened anxiety levels exhibit increased thigmotactic responses. At baseline and post-DI, there were no significant differences between the male Chow AL and the Chow EOD groups on the duration in the center arena (Fig. 7b). However, the male Chow AL group spent less time in the center arena post-DI when compared with baseline. This aging-related decline was not observed in the male Chow EOD group (Fig. 7b). Similar effects were also observed in the number of center arena entries (Fig. 7c). The latency to the center arena was unaffected by both the DI status and time in male mice (Fig. 7d). Furthermore, in female mice, there were no significant effects of the DI status or the time on the duration (Fig. 7b), the number of entries (Fig. 7c), and latency to the virtual center arena (Fig. 7d).

Rearing behavior, which is suggested to reflect reduced anxiety in mice [120], was maintained in the male Chow EOD group, but not in the Chow AL group (Fig. 7e–g). Specifically, similar to the thigmotactic response, there were no significant between-group differences on the rearing duration, rearing counts, and latency to rearing in males (Fig. 7e–g). However, there was an aging-related reduction in the rearing duration (Fig. 7e) and the rearing counts (Fig. 7f) in the male Chow AL group from baseline to post-DI. In contrast, this decline was not observed in the male Chow EOD group. In females, there was no effect of DI status on the rearing duration, rearing counts, and latency to rear at each time point (Fig. 7e–g); however, both the Chow AL and Chow EOD female had aging-related decreases in rearing duration (Fig. 7e) and counts (Fig. 7f) post-DI when compared with baseline.

The aging-related decline in ambulatory behavior was prevented by EOD fasting in male mice. At baseline and post-DI, the distance traveled/path length and the average velocity between the male Chow AL and the Chow EOD groups were not significantly different (Fig. 7h, i). However, there was a significant decrease in distance traveled and velocity from baseline to post-DI in the male Chow AL group that was not observed in the male EOD fasting group (Fig. 7h, i). Conversely, in females, there was no effect of DI or time points on ambulatory behavior (Supplemental Figure 7). Taken together, these findings indicate that late-life initiated EOD fasting prevents aging-induced anxiety-like behavior and declines in ambulatory activity in a sex-specific manner.

### Late-life EOD fasting enhances long-term object location memory

**Fig. 8:**
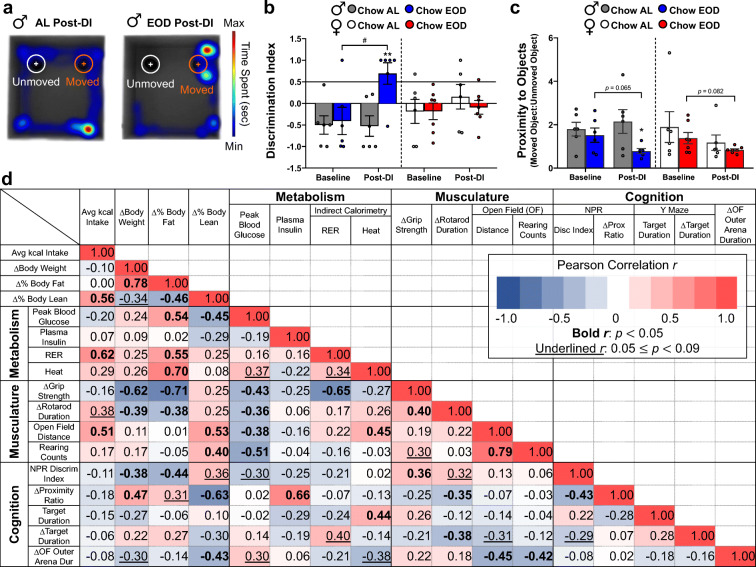
Late-life EOD fasting enhances long-term object location memory. **a** Representative heatmaps of post-DI performance in the novel pace recognition (NPR) task in the male Chow AL (*left*) and Chow EOD groups (*right*). **b**, **c** Long-term object location memory was tested at baseline and post-DI. Discrimination index at baseline and post-DI in the male (**b** *left*) and female (**b** *right*) Chow AL and Chow EOD groups (*n* = 6–7 mice per sex per group). Proximity to object, a ratio of the average distance from the nose of the mouse to the moved and unmoved objects measured at baseline and post-DI in the male (**c** left) and female (**c** right) Chow AL and Chow EOD groups. The figures depict mean with error bars (± SEM). The asterisks indicate the significant difference between the same-sex Chow AL and Chow EOD groups. **p < 0.01. The pound signs indicate the significant within group difference between the baseline and a post-DI time point. ^#^*p* < 0.05. **d** Correlation matrix showing the interrelationship among the selected metabolic, musculoskeletal, neurobehavioral, and cognitive measures from all of the mice in Pearson’s *r*. The bold *r* values indicate a significant correlation coefficient, *p* < 0.05. The underlined *r* values indicate a correlation coefficient, *p* < 0.09 but greater than 0.05

To test whether the memory-enhancing effects of late-life initiated EOD fasting on spatial memory are reproducible in another hippocampal-dependent memory task, we conducted the NPR test at baseline and post-DI in male and female mice. They were given a 5-min training trial on day 1 and a 2-min retention trial 24 hr later, with representative data output shown in Fig. 8a. Most mice, at least when young adults, prefer to investigate a novel location object [121]. In the baseline retention trial, we detected no significant difference between groups within sex (Fig. 8b). However, EOD fasting in males increased their discrimination index post-DI compared to the Chow AL group, reflecting an increase in the investigation of the object at a new location relative to the object that remained at the same location (Fig. 8b). When compared with their baseline discrimination index, the post-DI discrimination index of the male EOD fasting group was increased, specifically above 0.5, indicating that their hippocampal-dependent memory was enhanced following the DI (Fig. 8b). The EOD fasting-induced enhancement was similarly observed in the proximity to objects measure, which is a ratio of the average distance from the nose of the mouse to the moved and unmoved objects (Fig. 8c). Specifically, the male EOD group directed their face closer (i.e., investigation) to the novel location object more than the Chow AL group post-DI. Furthermore, although it was non-significant, a decreasing trend (*p* = 0.065) in the proximity to objects measure was observed compared to itself baseline/pre-DI (Fig. 8c). In contrast, in females, these memory-enhancing effects of EOD fasting were not observed. Specifically, there were no effects of DI or time points on the discrimination index (Fig. 8b). However, there was a decreasing trend (*p* = 0.082) in the proximity to objects measure in the female Chow EOD group post-DI when compared with baseline (Fig. 8c). Thus, the findings that EOD fasting increased the post-DI discrimination index and duration of novel object location investigation relative to (1) baseline and (2) Chow AL counterparts in males suggest that late-life initiated EOD fasting enhances hippocampal-dependent object placement memory in aged male mice.

### Aging-related frailty variables are inter-correlated in aged mice

Previously, Rockwood et al. (2017) [16] determined frailty by counting the number of deficits accumulated in aged, and with that, they found highly frail individuals or mice would have a decrease in multiple health and physiological conditions. Thus, we tested the a priori-predicted relationships between each frailty measure with the other frailty measures utilized in our current study on a per animal basis. Pearson’s correlation coefficients were computed among the selected metabolic, musculoskeletal, neurobehavioral, and cognitive measures collected from all of the mice (males and females combined) to produce a correlation matrix (Fig. 8d). In general, individual frailty variables co-present with other individual frailty variables, which is in line with previous studies using the frailty indices for mice [18]. Thus, our findings suggest that the aged mice who exhibit a deficit in one of the frailty variables are likely to perform poorly in other frailty measures, and EOD fasting thus dampens the onset and/or severity of multiple frailty endpoints.

### Renal H_2_S production capacity is enhanced by late-life EOD fasting and correlates with improved frailty measures

**Fig. 9:**
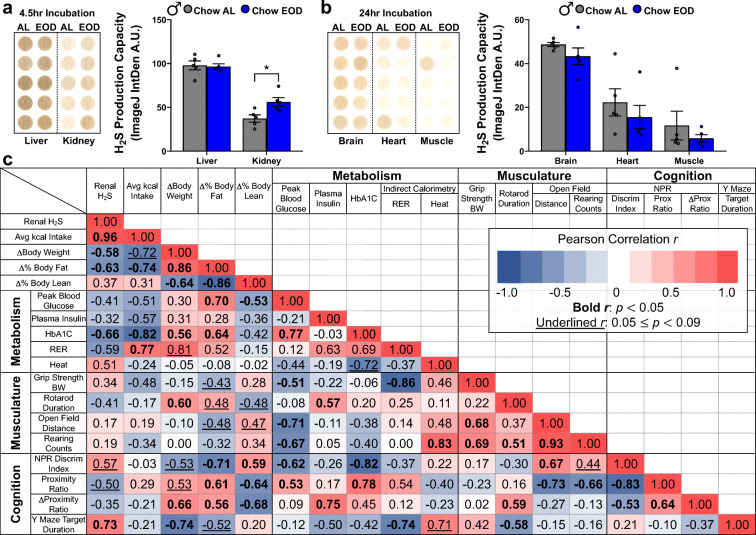
H_2_S production capacity is enhanced by late-life EOD fasting in a tissue-specific manner and correlates with improved frailty measures. **a**, **b** H_2_S production capacity in liver and kidney (**a**) and in brain, heart, and muscle (**b**) in the male Chow AL (*n* = 5) and Chow EOD groups (*n* = 5) measured by the filter paper-embedded lead acetate endpoint assay. Images with quantitated lead sulfide spots on the filter paper following a 4.5-hr incubation (**a**) and a 24-hr incubation (**b**). The figures depict the mean with error bars (± SEM). The asterisks indicate the significant difference between the male Chow AL and Chow EOD groups. *p < 0.05. **c** Correlation matrix showing the interrelationship among the renal H_2_S production capacity and the various frailty measures obtained from male mice in Pearson’s *r*. The bold *r* values indicate a significant correlation coefficient, *p* < 0.05. The underlined *r* values indicate a correlation coefficient, *p* < 0.09 but greater than 0.05. See also Supplemental Figure 9

Endogenous H_2_S production and its downstream signaling have gained ground as a biomarker [78, 80] and possible causal factor for increased healthspan and lifespan [96, 122–124]. Meanwhile, aging decreases H_2_S production in the liver and kidney [79, 125]. However, it is not clear whether late-life EOD fasting maintains and/or augments H_2_S production in a tissue-specific manner and whether the beneficial effects of late-life EOD fasting are associated with changes in H_2_S production capacity. Thus, we next tested the possible association of endogenous H_2_S production capacity on the beneficial anti-frailty effects of late-life EOD fasting that were demonstrated in the male mice. We first measured H_2_S production capacity in liver, kidney, heart, muscle (quadriceps), and brain from the male and female Chow AL and Chow EOD groups. Similar to previous findings in our laboratory [126, 127], we detected the highest endogenous H_2_S production capacity in the liver and kidney (Fig. 9a, Supplemental Figure 9A), while less H_2_S production was observed in the brain, heart, and muscle (Fig. 9b). EOD fasting in males increased H_2_S production capacity in the kidney (Fig. 9a); however, EOD fasting had no impact on the H_2_S production in the liver, brain, heart, and muscle (Fig. 9a, b). In females, there were no significant diet-induced changes in H_2_S production for any of the organs assayed (Supplemental Figure 9B, C). To rule out the possibility that the renal H_2_S production capacity was enhanced in the male Chow EOD group by reversing aging-related renal injury, rather than by an augmentation of renal H_2_S production capacity *per se*, we measured plasma creatinine levels in male mice. EOD fasting had no impact on circulating creatinine, as the plasma creatinine levels were equivalent between the Chow AL and the Chow EOD groups (Supplemental Figure 9D). These findings suggest that the effects of EOD fasting on H_2_S production capacity are tissue-specific and enhance renal H_2_S production.

Although correlation does not imply causation, it is necessary to conclude the dependency of an effect on a specific mechanism. Thus, as a first step, we computed a correlation matrix for renal H_2_S production capacity and the various frailty measures obtained from male mice only to discover whether significant relationships (Fig. 9c). There were significant correlations between renal H_2_S production capacity and improvements in metabolic and cognitive endpoints, but not with muscular function. However, the causal role of enhanced renal H_2_S production capacity in many of the anti-frailty benefits derived from the EOD fasting intervention is yet to be determined. Taken together, our finding shows that renal H_2_S may modulate the positive effects of EOD fasting on frailty, at least it correlates with the frailty improvements in males.

### Late-life EOD fasting affects neuroinflammatory cytokines and a neuronal antioxidant defense gene, but not the anorexigenic, orexigenic, and synaptic plasticity-related genes in the hypothalamus

**Fig. 10:**
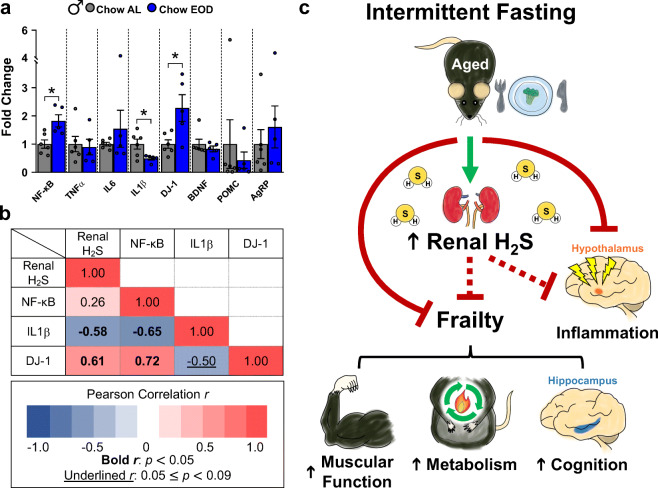
Late-life EOD fasting affects neuroinflammatory cytokines and a neuronal antioxidant defense gene, but not the anorexigenic, orexigenic, and synaptic plasticity-related genes in the hypothalamus. **a** The selected hypothalamic gene expression in the male Chow AL (*n* = 6) and Chow EOD groups (*n* = 5) measured by the real-time quantitative PCR (qPCR) analysis. The values are expressed as fold change relative to the male Chow AL group gene expression levels after normalizing each sample to β-actin using the ΔΔC_T_ method. The figure (**a**) depicts the mean with error bars (± SEM). The asterisks indicate the significant difference between the male Chow AL and Chow EOD groups. *p < 0.05. **b** Correlation matrix showing the interrelationship among the renal H_2_S production capacity and the selected hypothalamic gene expression in Pearson’s *r*. The bold *r* values indicate a significant correlation coefficient, *p* < 0.05. The underlined *r* values indicate a correlation coefficient, *p* < 0.09 but greater than 0.05. **c** Schematic diagram depicting the hypothesized relationship between intermittent fasting, aging-related frailty, and renal H_2_S production

The brain is a master regulator of energy intake and expenditure, muscular function, and cognition. Especially, the hypothalamus is a critical brain area for energy regulation and metabolism [128–130], and inflammatory responses in the hypothalamus contribute to obesity, glucose homeostasis dysregulation, and overall health maintenance [131]. Therefore, we examined hypothalamic gene expression profiles related to energy balance, inflammation, oxidative stress, and the synaptic plasticity of the male mice in our study. We found that expression of NF-κB, a master regulator of pro-inflammatory cytokine and survival gene expression [132], was increased by 1.82-fold in the EOD fasting group compared to the Chow AL group (Fig. 10a). However, downstream pro-inflammatory cytokines IL6 and TNF-α were unaffected by the EOD fasting. Interestingly, EOD fasting downregulated another downstream pro-inflammatory cytokine IL1β by 0.49-fold, indicating that the EOD fasting selectively affects NF-κB-associated downstream activity (Fig. 10a). Additionally, EOD fasting upregulated hypothalamic gene expression of DJ-1, a neuroprotective chaperone protein involved in inhibiting unwanted protein aggregation and sensing for oxidative stress [133], by 2.28-fold relative to the Chow AL group (Fig. 10a). In contrast, hypothalamic BDNF, POMC, and AgRP gene expressions were unaffected by the EOD fasting (Fig. 10a).

To further elucidate the relationship between renal H_2_S production capacity and the hypothalamic pro-inflammatory cytokine and antioxidant defense genes, we computed a correlation matrix (Fig. 10b). We found that renal H_2_S production was not associated with the hypothalamic NF-κB gene expression; however, the renal H_2_S production capacity was associated with a reduction in the IL1β gene expression and with increased expression of the DJ-1 gene (Fig. 10b). In sum, EOD fasting reduces hypothalamic inflammatory cytokine and increases neuroprotective gene expression, which are associated with renal H_2_S production capacity. Therefore, it raises the possibility that late-life EOD fasting slows neuroinflammation via boosting renal H_2_S production capacity.

## Discussion

The present study is the first to show that late-life initiated EOD intermittent fasting decreases aging-related frailty in a sexually dimorphic manner, and the improvements in frailty correlate with renal H_2_S production. Specifically, the present study showed that late-life initiated EOD fasting in 20-month-old mice decreased overall energy intake in males, but not in females. Also, EOD fasting reduced body weight and fat mass and enhanced metabolic fitness, muscular functions, and hippocampal-dependent memory in males, but few positive effects of EOD fasting on these measures were observed in females. Furthermore, we demonstrated that EOD fasting affected hypothalamic inflammatory responses and increased renal H_2_S production in males. Interestingly, the renal H_2_S production capacity was positively correlated with improvements in aging-related frailty. Collectively, these results suggest that late-life initiated EOD fasting attenuates multiple components of aging-related frailty in a sex-dependent manner and that these frailty improvements may be due to increased production of renal H_2_S production, as summarized in Fig. 10c.

Several lines of evidence suggest that the present finding of the sexually dimorphic effects of EOD fasting may be due to the difference in the overall caloric intake between males and females. Specifically, we showed that late-life EOD fasting increased caloric intake during the fed days in both male and female mice. However, female mice consumed almost twice as much food during the fed periods compared to the controls, resulting in comparable food intake overall to that of the control/AL group. In contrast, there was an overall reduction of caloric intake, specifically by 30–40%, in the male EOD fasting group compared to the controls, which may have been a driving force for the beneficial outcomes, as CR of this amount has previously shown to improve multiple aging-related outcomes. For example, 10–40% CR lowered circulating pro-inflammatory cytokines [134] and enhanced glucose homeostasis in the same strain of mice as the present study [134, 135]. Conversely, it is also possible that the outcomes of EOD fasting in females, which was equivalent to the time-restricted feeding without decreasing overall kcal intake, produced a somewhat lesser degree of improvement. In support of this prediction, a similar effect was observed in the study by Anson et al. [75]. Specifically, the EOD fasting regimen in the same strain of male mice did not affect the overall food intake due to consuming twice as much of food as the controls following the 24-hr fasting period. As a result, they also showed that the effect of EOD fasting was somewhat dampened compared with the continuous CR group. Both the EOD fasting and the continuous CR enhanced glucose metabolism/insulin sensitivity; however, the levels of circulating insulin-like growth factor-1 (IGF-1) were oppositely affected by these dietary regimens [75]. Similarly, the same research group showed that the effects of EOD fasting on behavior and its associated molecular change were slightly attenuated when compared with the continuous CR group. Specifically, both the EOD fasting and the CR decreased aging-related neurobehavioral deficits (i.e., decreased locomotion and memory impairments); however, EOD fasting did not decrease hippocampal amyloid β-peptide, while CR did [76]. Therefore, the relatively isocaloric EOD fasting compared with the AL feeding observed in the female mice in our current study may have lessened the beneficial effects of EOD fasting. This prediction is likely given that the comparative mice study by Mitchell et al (2019) [64] showed that the 30 % CR group experienced the most prominent anti-aging effect on metabolic flexibility, insulin sensitivity, lifespan, and pathological changes compared with the time-restricted feeding group, which was isocalorically fed to that of the control/AL feeding group (similar to our EOD fasting in females). On the other hand, the time-restricted feeding group gained some benefits similar to that of the 30% CR group, but to a lesser degree [64].

It is also possible that the sexually dimorphic effect of EOD fasting was due to the sex-dependent frailty responses to aging. In general, there is a higher prevalence of aging-related frailty in female humans [136–140] and other mammals ([18]; e.g., mice [10, 14, 141, 142] and dogs [143]). The present finding did not make a direct comparison between male and female mice on the frailty levels due to the different food intake patterns during the DI period. However, it is possible that the beneficial effects of EOD fasting were not observed in females because the “dose” of the treatment (i.e., the stringency and/or duration of the caloric intake in EOD fasting) was not as effective in females as it was in males to reverse or rejuvenate aging-related frailty. Similar sex- and CR dose-dependent phenomena have been reported previously in mice undergoing CR and other dietary restrictions, such as protein restriction [144, 145]. For instance, Mitchell et al. (2016) [144] showed that 20–40% CR affected lifespan, glucose homeostasis, lifespan, body core temperature, aging-related pathological changes, hepatic gene expression, and hepatic H_2_S production capacity to a different degree for each sex [144]. Specifically, 20–40% CR produced overall improvements in both male and female mice; however, for example, 40% CR induced greater hepatic H_2_S production than the 20% CR in females, but 20 and 40% CR produced comparable hepatic H_2_S in males [144]. Similarly, in another study [146], female mice responded to both high and low doses of rapamycin, a longevity-associated drug/pharmacological diet restriction mimetic, while male mice did not positively respond to the low dose in regard to survival. Furthermore, acarbose, 17-α-estradiol, nordihydroguaiaretic acid, and methylene blue, all of which are implicated as anti-aging agents, produced sex-dependent responses in survivorship, glucose metabolism, and activity levels [147]. Alternatively, our sex-dependent EOD fasting outcomes could be attributed to differential maintenance of reproductive versus survival programs in males and females while under dietary stress [145].

In a more simplistic explanation for the differences in male and female responses to EOD fasting, it may be possible females at 20 months of this age in our study are not yet having severe aging phenotypes like males, and that could explain why differences in AL vs. EOD is smaller in females compared to those males at this age. Perhaps a longer duration of EOD fasting may lead to a greater differential in the parameters measured. However, the caveat of waiting too long is the potential for survival bias at very old ages in mice beyond 3 years, in the sense that mice will either be very sick or selected for resiliency. This is one main reason for the choice of aging time point we utilized to perform the dietary intervention and frailty tests (20 months of age), as it is relatively safe and prevents survival bias. So while it is not apparent the mice were showing hallmarks of aging at the start of the study, at the very least the AL fed male mice showed the signs of aging during the study that were somewhat dampened in females.

Previously established and validated frailty indices for rodents (i.e., deficit accumulation model [148]) estimate frailty using various behavioral and physiological assessment tools [11–15]. However, others [18] emphasize that one of the limitations of these studies is the lack of cognitive tasks. To circumvent this disadvantage, and given that the prevalence of memory deficits and associated Alzheimer’s disease remain high in older adults (70+ years; [149]), we assessed the impact of aging and EOD fasting on hippocampal-dependent spatial memory, using the Y maze forced alternation task and the NPR task. Specifically, we found that in the forced alternation task, there was a significant, but minimal, spatial memory improvement in males induced by EOD fasting, but not in females (Fig. 9). Furthermore, we also found that EOD fasting enhanced object location memory in males, but only the tendency was observed in females (Fig. 8). These memory improvements were in line with previous DR studies in which DR (1) improves memory in adults [150] and older adults [45], (2) protects against *hippocampal*-dependent memory deficits and accumulations of hippocampal amyloid β-peptide plaques and neurofibrillary tangles in Alzheimer’s disease model of mice [76], and (3) rejuvenates hippocampal transcriptome profiles in middle-aged mice [151]. Additionally, similar to our present findings, Li et al. (2013) [73] showed that 11-month long EOD fasting in CD-1 wild-type male mice produced time-dependent memory enhancements. Specifically, EOD fasting enhanced memory that was tested 1 week after the last training trial (1-week inter-trial interval [ITI]) but did not enhance memory when tested 24 hr after the last training trial (24 hr ITI). In other words, the retention trial occurred 24 hr after was not long enough to detect the memory retrieval deficits in their study [73]. In our study, a probe trial for the Y maze forced alternation task was given 2 hr after the acquisition trial, and the retention trial for the NPR task occurred 24 hr later. Therefore, it is possible that similar to the findings by Li et al. (2013) [73], the beneficial effects of EOD fasting on spatial memory improvement may have become more apparent if we utilized a longer retention ITI, especially for females.

We hypothesize that the beneficial effects of late-life initiated EOD fasting depend on the ability of the kidney to produce H_2_S. The kidney is one of the organs that produces high levels of H_2_S [127, 152, 153], which is extremely susceptible to the effects of aging. Specifically, aging decreases expression and/or activity of cystathionine γ-lyase (CGL) and cystathionine β-synthase (CBS), the H_2_S-generating enzymes [125, 154], which are rescuable by the exogenous H_2_S treatment [125, 154] or long-term CR [79]. A rat model of chronic kidney disease (CKD) downregulates CGL, CBS, and the subsequent H_2_S production in liver and kidney [155]. One of the associated symptoms observed in the older patients with CKD is memory deficits [156, 157], along with structural changes in brain morphology, such as the decreased size of the hippocampus [157]. Interestingly, in a rat model of CKD, a H_2_S donor, sodium hydrosulfide (NaHS) treatment decreased CKD-associated memory deficits [158]. These findings suggest that there is a kidney-brain (i.e., cognition) connection via H_2_S signaling. Furthermore, DR in young mice and aged rats [96, 159] increase H_2_S production in major metabolic organs, including the kidney. In the present study, we showed that EOD fasting in males improved frailty, which correlated positively with H_2_S production capacity in the kidney. The present findings did not reveal causation; however, it certainly showed the relationship between these two factors. Interestingly, we also observed that there was no enhanced renal H_2_S production capacity in females (Supplemental Figure 9B). This suggests that limited beneficial effects of EOD fasting in females may be due to the lack of EOD fasting-induced renal H_2_S production. Future molecular and genetic studies related to EOD fasting-induced alterations in tissue-specific protein sulfhydromes and the dependence of H_2_S generating enzymes for the late-life benefits of dietary interventions are still needed to test these theories.

In summary, our studies demonstrate for the first time that late-life initiated EOD fasting reduces frailty in aged mice, an effect that was somewhat dampened in the female mice. Importantly, our findings show that late-life EOD fasting attenuates aging-related cognitive decline, which is not typically included in the frailty assessment for rodents. The finding that frailty measures that were utilized in the present study were inter-correlated suggests the validity of these tools as aging-related frailty measures in mice. We also made the novel discovery that late-life EOD fasting of short duration near the end of a mouse’s life is sufficient to increase longevity-associate gasotransmitter H_2_S production in the kidney. Importantly, this increased renal H_2_S production was positively correlated with multiple improvements in frailty, as well as a reduction in the inflammatory response in the hypothalamus. Our current work, combined with a growing field of study for mid-life to late-life dietary interventions such as IF and time-restricted feeding [59, 160], offer potential therapeutic avenues and approaches to improve healthspan and prevent cognitive impairment. This emphasizes that it may never be too late to slow or reverse the ravages of aging with lifestyle interventions. Given the relative feasibility of integrating an EOD fasting regimen into the human lifestyle, particularly in older adults, and the beneficial effects of H_2_S at the appropriate concentration as an anti-aging agent, our findings suggest that this dietary regimen may be particularly useful for the older adult population to promote a healthy lifespan.

## Supplementary Information

ESM 1 (XLSX 27 kb)

ESM 2 (XLSX 269 kb)

ESM 3 (XLSX 19 kb)

ESM 4 (XLSX 14 kb)

ESM 5 (XLSX 13 kb)

ESM 6 (XLSX 21 kb)

ESM 7 (XLSX 19 kb)

ESM 8 (XLSX 1330 kb)

ESM 9 (XLSX 13 kb)

ESM 10 (XLSX 11 kb)

ESM 11 (XLSX 9 kb)

ESM 12 (XLSX 13 kb)

ESM 13 (PDF 908 kb)
