# Supplementary material for: Late-life intermittent fasting decreases aging-related frailty and increases renal hydrogen sulfide production in a sexually dimorphic manner
Source: GeroScience. 2021 Mar 6;43(4):1527–54. doi: 10.1007/s11357-021-00330-4 (PMC8492807; doi:10.1007/s11357-021-00330-4)
Supplement: Supplementary file 13 — (PDF 908 kb) [file 11357_2021_330_MOESM13_ESM.pdf]

Supplemental Data Figures and their Legends for both review purposes and intended for publication as an online data supplement for the manuscript titled "**Late-life intermittent fasting decreases aging-related frailty and increases renal hydrogen sulfide production in a sexually dimorphic manner**" by Yoko O. Henderson, Nazmin Bithi, Christopher Link, Jie Yang, Rebecca Schugar, Natalia Llarena, J. Mark Brown, and Christopher Hine

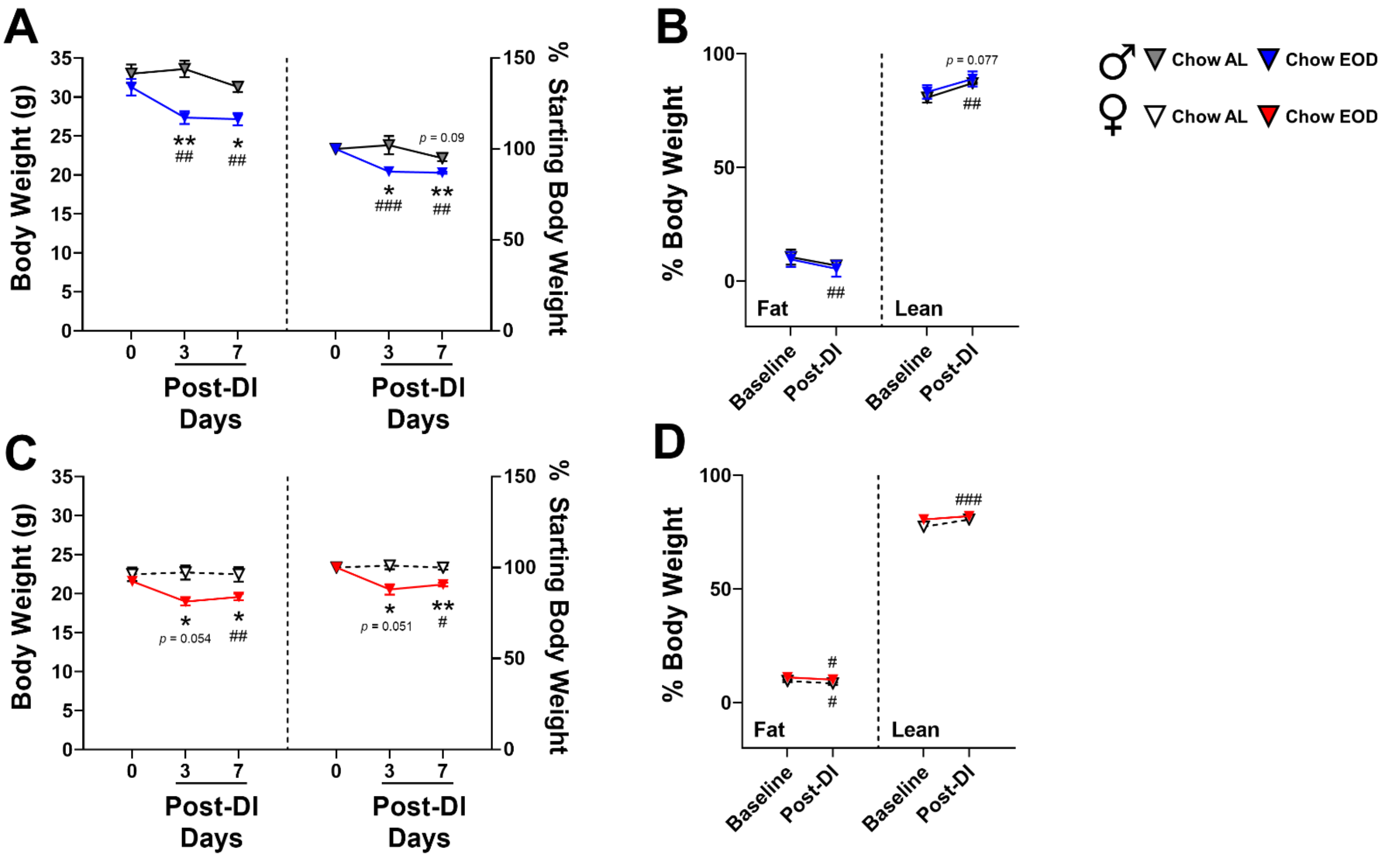

Supplemental Figure 1: EOD fasting decreases body weight and alters body composition in a sexually dimorphic manner in young adult mice. (**A-D**) Body mass and body composition in 6-month old male and female mice on Chow AL and Chow EOD feeding. Absolute body weight (*left*) and % body weight compared with baseline (*right*) in the male (**A**) and female (**C**) Chow AL and Chow EOD groups from baseline to post-dietary intervention (post-DI). (**B, D**) % fat mass adjusted to body weight (*left*) and % lean mass adjusted to body weight (*right*) in the male (**B**) and female (**D**) Chow AL and Chow EOD groups at baseline and post-DI. The figures depict the mean with error bars (± SEM) and n = 3 mice/diet group per sex. The asterisks indicate the significant difference between the same-sex Chow AL and Chow EOD groups. *$p < 0.05$, **$p < 0.01$, ***$p < 0.001$, and ****$p < 0.0001$. The pound signs indicate the significant within group difference between the baseline and a post-DI time point. $^{\#}p < 0.05$, $^{\#\#}p < 0.01$, $^{\#\#\#}p < 0.001$, and $^{\#\#\#\#}p < 0.0001$. See also Figure 1.

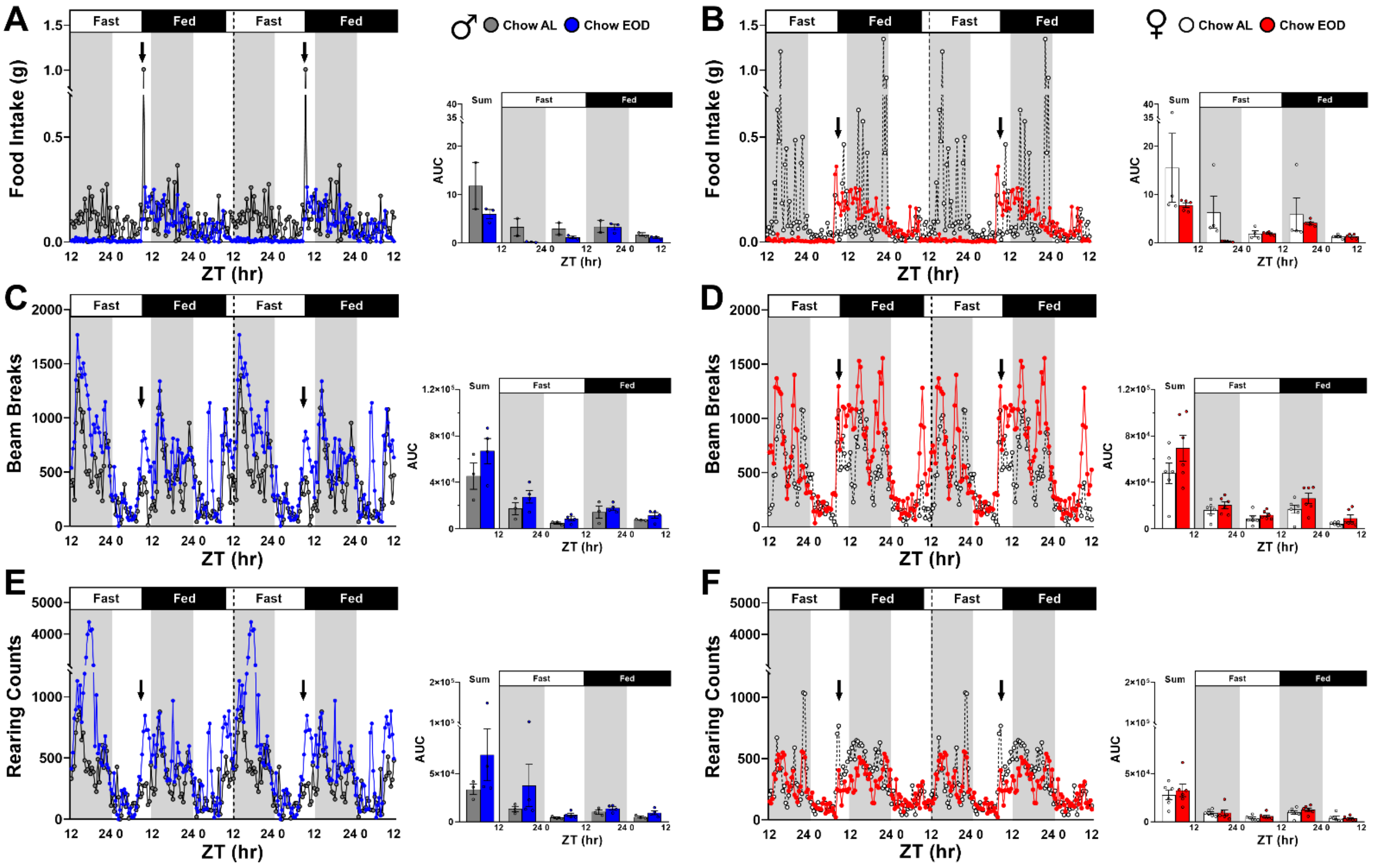

Supplemental Figure 2: Late-life EOD fasting does not affect circadian patterns of food intake, ambulation, or rearing. (**A-F**) Food intake (g) per mouse, beam breaks, and rearing counts over the 4-day period in the metabolic chamber were measured approximately every 20 min in the male (**A, C, E**) and female (**B, D, F**) Chow AL and Chow EOD groups (n = 3-6 mice per sex per group). The figures (**A-F**) depict the mean with no error bars for a representation purpose, while the insets show the AUC for combined/summation of area, fast day 12:12 light:dark cycle, and fed day 12:12 light:dark cycle for each measure. The inset figures depict the mean with error bars (± SEM). See also Figure 2.

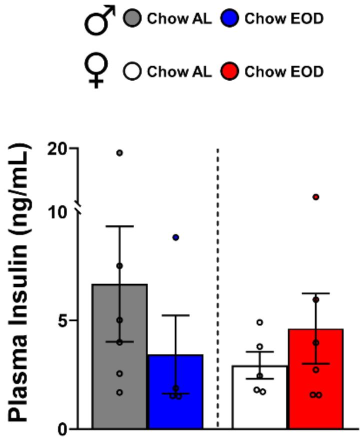

Supplemental Figure 4: Late-life EOD fasting did not affect circulating insulin levels. Plasma insulin levels (ng/mL) in the male (*left*) and female (*right*) Chow AL and Chow EOD groups (n = 4-6 mice per sex per group). The data depict the mean with error bars (± SEM). See also Figure 4.

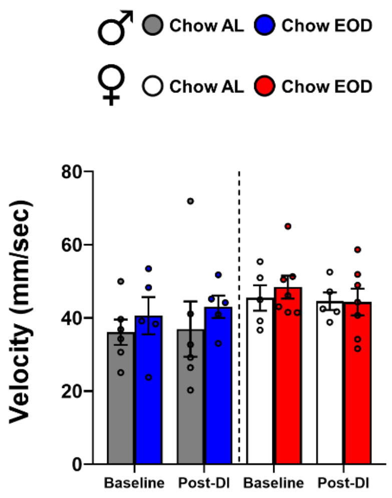

Supplemental Figure 6: Late-life EOD fasting does not affect locomotion during the hippocampal-dependent short-term memory test. Velocity in the Y Maze forced alternation task at baseline and post-DI in the male (*left*) and female (*left*) Chow AL and Chow EOD groups (n = 5-7 mice per sex per group). The figure depicts the mean with error bars (± SEM). See also Figure 6.

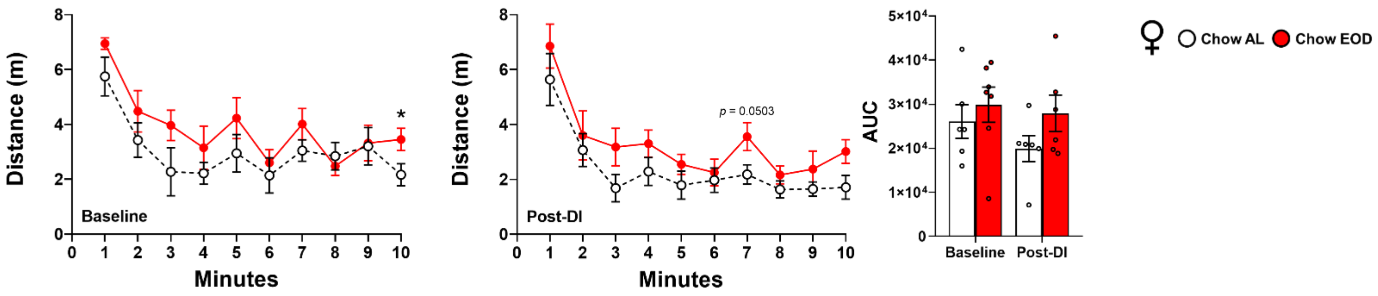

Supplemental Figure 7: Late-life EOD fasting does not affect motor activity in females during the open field task. Velocity in the open field at baseline and post-DI in the female Chow AL (n = 6) and Chow EOD groups (n = 6). The figure depicts the mean with error bars (± SEM). The asterisks indicate the significant difference between the same-sex Chow AL and Chow EOD groups. *$p < 0.05$. See also Figure 7.

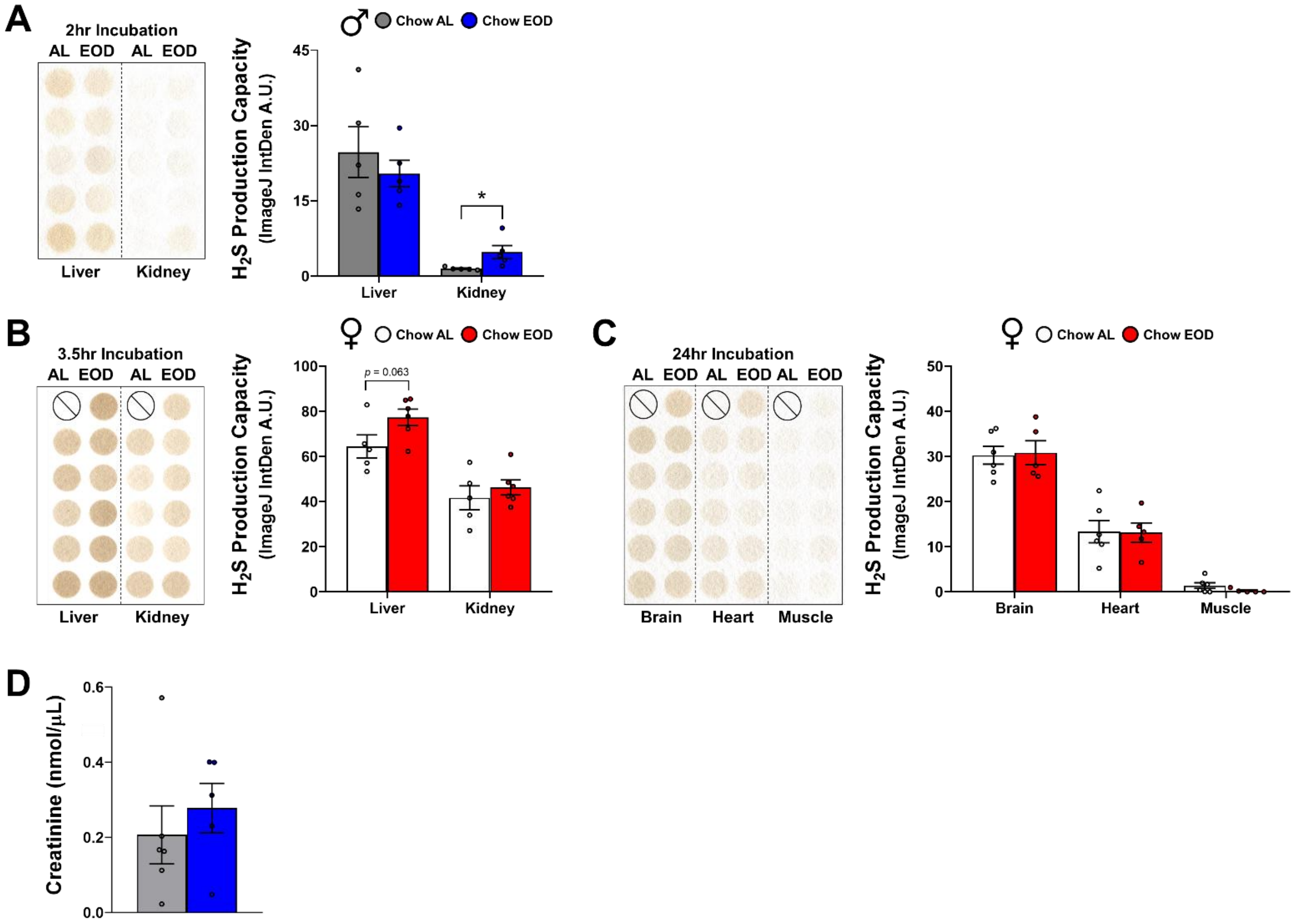

Supplemental Figure 9: Renal H_2S production capacity is enhanced by late-life EOD fasting in a sex-specific manner and without affecting circulating plasma creatinine levels. (**A**) H_2S production capacity in liver and kidney in the male Chow AL (n = 5) and Chow EOD groups (n = 5). Images with quantitated lead sulfide spots on the filter paper following a 2-hr incubation. (**B, C**). H_2S production capacity in liver and kidney (**B**) and in brain, heart, and muscle (**C**) in the female Chow AL (n = 5) and Chow EOD groups (n = 6). Images with quantitated lead sulfide spots on the filter paper following a 3.5-hr incubation (**B**) and a 24-hr incubation (**C**). (**D**) Plasma creatinine levels in the male Chow AL (n = 6) and Chow EOD groups (n = 5). The figures (**A-D**) depict the mean with error bars (± SEM). The asterisks indicate the significant difference between the male Chow AL and Chow EOD groups. *$p < 0.05$. See also Figure 9.
